# TIPE Regulates DcR3 Expression and Function by Activating the PI3K/AKT Signaling Pathway in CRC

**DOI:** 10.3389/fonc.2020.623048

**Published:** 2021-02-24

**Authors:** Mengya Zhong, Xingfeng Qiu, Yu Liu, Yan Yang, Lei Gu, Chenxi Wang, Huiyu Chen, Zhongchen Liu, Jiayin Miao, Guohong Zhuang

**Affiliations:** ^1^ Cancer Research Center, School of Medicine, Xiamen University, Xiamen, China; ^2^ Department of Hematology, The First Affiliated Hospital of Xiamen University and Institute of Hematology, School of Medicine, Xiamen University, Xiamen, China; ^3^ Department of Gastrointestinal Surgery, Zhongshan Hospital, Xiamen University, Xiamen, China; ^4^ General Surgery Center of Bazhong Central Hospital, Bazhong, China; ^5^ Department of Gastrointestinal Surgery, Shanghai Tenth People’s Hospital Affiliated to Tongji University, Shanghai, China; ^6^ Department of Neurology, Zhongshan Hospital, Xiamen University, Xiamen, China; ^7^ Organ Transplantation Institute of Xiamen University, Fujian Provincial Key Laboratory of Organ and Tissue Regeneration, School of Medicine, Xiamen University, Xiamen, China

**Keywords:** colorectal cancer, cell proliferation, migration, apoptosis, PI3K/AKT signaling pathway

## Abstract

Tumor necrosis factor-induced protein-8 (TIPE) is highly expressed in colorectal cancer (CRC). Decoy receptor 3 (DcR3) is a soluble secreted protein that can antagonize Fas ligand (FasL)-induced apoptosis and promote tumorigenesis. It remains unclear whether TIPE can regulate DcR3 expression. In this study, we examined this question by analyzing the relationship between these factors in CRC. Bioinformatics and tissue microarrays were used to determine the expression of TIPE and DcR3 and their correlation in CRC. The expression of TIPE and DcR3 in colon cancer cells was detected. Plasma samples were collected from CRC patients, and DcR3 secretion was measured. Then, dual-luciferase reporter gene analysis was performed to assess the interaction between TIPE and DcR3. We exogenously altered TIPE expression and analyzed its function and influence on DcR3 secretion. Lipopolysaccharide (LPS) was used to stimulate TIPE-overexpressing HCT116 cells, and alterations in signaling pathways were detected. Additionally, inhibitors were used to confirm molecular mechanisms. We found that TIPE and DcR3 were highly expressed in CRC patients and that their expression levels were positively correlated. DcR3 was highly expressed in the plasma of cancer patients. We confirmed that TIPE and DcR3 were highly expressed in HCT116 cells. TIPE overexpression enhanced the transcriptional activity of the DcR3 promoter. TIPE activated the PI3K/AKT signaling pathway to regulate the expression of DcR3, thereby promoting cell proliferation and migration and inhibiting apoptosis. In summary, TIPE and DcR3 are highly expressed in CRC, and both proteins are associated with poor prognosis. TIPE regulates DcR3 expression by activating the PI3K/AKT signaling pathway in CRC, thus promoting cell proliferation and migration and inhibiting apoptosis. These findings may have clinical significance and promise for applications in the treatment or prognostication of CRC.

## Introduction

Colorectal cancer (CRC) kills nearly 2 million people each year, making it the fourth deadliest cancer worldwide behind lung, liver and stomach cancers (1). According to The Global Cancer Observatory (GLOBOCAN) 2018 data (http://gco.iarc.fr), CRC is currently the most common malignant tumor in China, ranking first in prevalence and second in incidence. Surgery, chemotherapy, radiotherapy and molecular targeted therapy are the most commonly used treatments for CRC, but the survival rate remains low (2). Patients with advanced CRC who receive radiation therapy and chemotherapy experience serious adverse reactions. Therefore, the development of new, effective treatment strategies is urgently needed. Currently, researchers are devoted to elucidating the molecular mechanism of the occurrence and development of CRC (3).

Tumor necrosis factor-induced protein-8 (TNFAIP8/TIPE; also called SCC-S2, MDC-3.13, GG2-1, and NDED) was the first identified protein in the TIPE family and is closely associated with tumors and inflammation (4). As an antiapoptotic and carcinogenic molecule, TIPE promotes the growth, proliferation and migration of cancer cells (5). The activation of TNF-α and NF-κB in the inflammatory environment can induce TIPE expression (6). A large number of studies have revealed that TIPE is highly expressed in various tumors, and high TIPE expression is related to clinical parameters and metastasis (7–9). TIPE has been reported to be overexpressed in colon cancer and to regulate cell proliferation (10). The latest research shows that TIPE can promote tumor proliferation and invasion by activating Wnt signaling and inhibiting Hippo signaling in CRC cells (11). Our previous studies showed that TIPE promotes angiogenesis in CRC by regulating VEGFR2 expression.

Decoy receptor 3 (DcR3) is a member of the tumor necrosis factor receptor superfamily (TNFRSF). DcR3 cDNA encodes the 300-amino acid (aa) protein, which contains four cysteine-rich repeats of TNFRSF. Unlike most members of the TNFRSF, DcR3 is a soluble secreted protein lacking transmembrane sequences and can be detected in serum and cell culture media (12). DcR3 binds and neutralizes three members of the tumor necrosis factor superfamily (TNFSF): Fas ligand (FasL) (13), TNF-like molecule 1A (TL1A) (14), and herpes virus entry mediator L (LIGHT) (15). By binding these ligands, DcR3 can inhibit apoptosis, induce angiogenesis and modulate immune cell function. Additionally, DcR3 is almost undetectable in most individuals with noninflammatory diseases and cancers. The expression level of DcR3 protein is related to tumorigenesis and metastasis (16). Earlier studies confirmed that DcR3 is a predictor of 5-fluorouracil (5-FU)-based adjuvant chemotherapy responses in CRC patients (17). Later, Yu et al. confirmed that DcR3 has the potential to regulate the growth and metastasis of SW480 colon cancer cells (18). Zong et al. found that the overexpression of DcR3 in CRC increases the risk of malignancy (19). These findings suggest that DcR3 is a potential therapeutic target in CRC.

Our previous studies demonstrated that TIPE is highly expressed in stage III gastric cancer and positively correlated with DcR3 and ERK1/2 (20). However, it is unclear whether TIPE can regulate the expression of DcR3; both of these factors can act as antiapoptotic molecules to antagonize cell apoptosis and promote cell proliferation and metastasis. In this study, with bioinformatic methods, we first detected high TIPE and DcR3 expression in CRC and found a positive correlation between the expression of these two proteins. In addition, we collected plasma samples from CRC patients diagnosed at the Department of Gastrointestinal Surgery at Zhongshan Hospital of Xiamen University, and high DcR3 expression was detected in the plasma. Dual-luciferase reporter gene analysis showed that TIPE overexpression enhanced the transcriptional activity of the DcR3 promoter. Exogenous changes in the expression of TIPE in HCT116 cells also altered the expression of DcR3. *In vitro* functional experiments indicated that TIPE plays a vital role in the proliferation, migration and apoptosis of CRC cells. Interestingly, when we stimulated TIPE-overexpressing HCT116 cells with LPS, we found that the expression of phosphorylated AKT, P105, and P65 was upregulated compared to that in control cells. After using the PI3K inhibitor LY294002 to suppress upstream PI3K expression, the abovementioned molecular phosphorylation was downregulated. Finally, these data combined with tissue microarray data demonstrated that TIPE and DcR3 were highly expressed in CRC and associated with poor prognosis. Our study revealed that TIPE regulated DcR3 expression by activating the PI3K/AKT signaling pathway in CRC, thereby promoting cell proliferation and metastasis and inhibiting apoptosis.

## Materials and Methods

### Cell Culture

The CRC cell lines HCT116, and SW620 and human embryonic kidney (HEK) 293T cells were obtained from the Anti-cancer Center of Xiamen University (Xiamen, Fujian). All cell lines were cultured in Dulbecco’s modified Eagle’s medium (DMEM, Gibco, CA, USA) supplemented with 10% fetal bovine serum (FBS, Gibco), 100 units/ml penicillin and 100 mg/ml streptomycin (Invitrogen, Carlsbad, CA, USA) and in a humidified environment containing 5% CO_2_ at 37°C.

### Cell Transfection

The lentiviral vector and the control vector (TIPE/PLNX2) encoding TIPE were donated by the School of Life Sciences, Xiamen University. We used 293T and HCT116 cells in the logarithmic growth phase to perform experiments. The day before transfection, 1×10^6^ cells were seeded into 6-well plates, and 2 ml serum-free medium was added to each well. According to the manufacturer’s instructions, 6 μl Lipofectamine 2000 (Invitrogen, Carlsbad, CA, USA) and 2 μg plasmid DNA were separately added to 250 μl Opti-MEM I serum-free medium (Sigma-Aldrich, St. Louis, USA) and incubated for 5 min, and then the two solutions were mixed and incubated at room temperature for 20 min. The mixture was added to the cells for transient transfection for 36 h. In all experiments, real-time quantitative PCR (qRT-PCR) and Western blot analysis showed that the transfection efficiency of the cells was > 60%. TIPE-overexpressing cells and the corresponding control cells were cultured for subsequent experiments.

### Cell Proliferation

According to the manufacturer’s instructions, cell proliferation activity was measured using a Cell Counting Kit-8 (CCK-8; TransGen Biotech, Beijing, China). Cells were seeded into 96-well plates (Corning Inc., NY, USA) in triplicate at an initial density of 4,000–7,000 cells per well and cultured in a 37°C, 5% CO2 incubator for 24 h. On the second day, CCK-8 reagent (10% of the total volume of medium) was added to each well, and the cells were cultured in the incubator for another 4 h. Finally, a Bio-Rad microplate reader (California, USA) was used to measure the absorbance at 450 nm at each indicated time point.

### Wound Healing Assay

In a six-well plate, lines were evenly drawn across the wells every 0.5–1 cm, with a total of three lines in each well. Transfected HCT116 cells (approximately 5×10^5^ cells per well) were added and cultured overnight. The next day, a 20-μl pipette tip was used to make a scratch as close to perpendicular to the horizontal line on the back as possible. Then, the cells were washed three times with preheated phosphate-buffered saline (PBS; Solarbio, Beijing, China) to detached cells, and 2 ml serum-free DMEM was added to each well. The cells were placed in a 37°C, 5% CO2 incubator, and measurements were taken after 0, 12, 24, and 36 h. ImageJ (NIH, MD, USA) was used to measure the scratch area. The experiment was repeated three times for each group of cells.

### Transwell Assay

HCT116 cells transfected with TIPE overexpression plasmids or TIPE interference plasmids (experimental group) and HCT116 cells transfected with PLNCX-2 empty vector plasmids (control group) were cultured in 6-well plates (5×10^5^ cells/ml) for 24 h, and then the cell medium was changed to serum-free DMEM. Cells were starved for 24 h. The two groups of cells were detached from the plates and counted, and a 2×10^5^ cell suspension was placed into a Transwell chamber. In addition, 500 μl of medium containing 15% FBS was added to the lower chamber, and the cells were cultured for 24 h. The Transwell chamber was removed, and the medium was discarded. The cells were washed three times with prechilled PBS, fixed with 4% paraformaldehyde for 20 min, and stained with 0.1% crystal violet for 15 min. Nonmigrated cells were gently removed from the top of the membrane with cotton swabs, and then the remaining invaded cells were observed and counted in 5 fields under a microscope at 100-fold magnification.

### Enzyme-Linked Immunosorbent Assay

Plasma from patients diagnosed with CRC was obtained from the Department of Gastrointestinal Surgery, Zhongshan Hospital Affiliated to Xiamen University. All aspects of the study were approved by the medical ethics committee of Zhongshan Hospital, Xiamen University. All registered patients provided written informed consent, and the use of patient samples in the study was approved by the Institutional Review Board of the Tumor Tissue Bank of Zhongshan Hospital, Xiamen University. Cell culture supernatant was obtained after cultivating cells for 24 h. Changes in DcR3 levels in cell supernatants and plasma were measured using an ELISA kit (R&D Systems, Minnesota, USA). Test samples and standards were added to a 96-well plate coated with the capture antibody, incubated at room temperature for 2 h and removed. The wells were washed with a series of buffers. Then, a biotin anti-human DCR3 antibody was added and incubated at room temperature for 1 h. The plate was washed three times, and an avidin-peroxidase complex was added and allowed to react for 30 min. After washing, tetramethyl benzidine (TMB) color solution was added, and the mixture was incubated at room temperature for 30 min in the dark. Finally, TMB stop solution was added, and the color in the wells changed. We determined the optical density (OD) value at 450 nm and calculated the corresponding concentration according to the absorbance value of the sample on the standard curve.

### Dual-Luciferase Reporter Gene Assay

The medium was removed from the cells, and the cells were washed with PBS. The wash solution was removed after washing. Then, 50 μl Report Lysis Buffer (Promega, Madison, Wisconsin, USA) was added to each well, the culture plate was gently shaken at room temperature for 15 min, the cells were scraped off the petri dish, and the sample was centrifuged at 16,000 g and placed at 4°C for 30 s. LARII (100 μl) was added to the test tube in advance, the program for the fluorescence luminometer was set, and the lysate was transferred to the test tube. Cell lysates containing an equal amount of protein (10–20 µg) were placed into the wells of an opaque black 96-well microtitration plate, 5 μl of luciferase substrate (Promega) was added, and firefly luciferase activity was detected on a Packard apparatus. Then, 100 μl Stop&Glo Reagent was added to the test tube to detect Renilla luciferase activity. Both firefly and Renilla luciferase activities were quantified according to the manufacturer’s instructions using the dual-luciferase reporter system (Promega).

### Western Blot Analysis

Cells were lysed with RIPA buffer (Sigma-Aldrich) at 4°C for 1 h with 1% protease inhibitor mixture and 1% phenylmethanesulfonyl fluoride (Gold Biotechnology, USA). The tricarboxylic acid (TCA) precipitation method was used to collect the supernatant of the culture medium to extract DcR3 protein. After centrifugation at 12,000 rpm for 10 min at 4°C, the supernatant was collected. The protein standard curve was developed by the BCA method, and protein concentrations of the samples were determined (Bio-Rad, Hercules, CA). The samples were mixed with sodium dodecyl sulfate (SDS) loading buffer, heated at 100°C for 10 min, and centrifuged at 12,000 rpm for 5 min. Then, an equal amount of protein (10–40 µg) was separated by electrophoresis on a 12% SDS gel, transferred to a PVDF membrane (Millipore, Billerica, MA, USA) and blocked with 5% skim milk powder. The membranes were washed and incubated at 4°C overnight with the following specific primary antibodies: rabbit monoclonal antibodies against TIPE (1:1,000; Abcam, MA, USA), PI3K, P-PI3K (1:1,000; Abcam, MA, USA), and P-AKT (1:2,000; Cell Signaling, MA, USA); a mouse monoclonal antibody against AKT (1:2,000; Cell Signaling, MA, USA); and rabbit polyclonal antibodies against DcR3 (1:1,000; Affinity Biosciences, Jiangsu, China), P105, P-P105, P65, P-P65 (1:500; Affinity Biosciences, Jiangsu, China), and β-actin (1:5,000; Proteintech, Suite, USA). The next day, immunoblotting was performed using the corresponding HRP-binding secondary antibody, followed by the visual detection of the protein bands using a hypersensitive enhance chemiluminescence (ECL) kit (NCM Biotech, Suzhou, China) and a Bio-Rad ChemiDoc XRS + detection system (Bio-Rad, Hercules, CA).

### Real-Time Quantitative PCR

Total RNA was extracted from cells using TRIzol reagent (TransGen Biotech, Beijing). One microgram of RNA was reverse transcribed into cDNA using a cDNA Synthesis SuperMix kit (TransGen Biotech, Beijing). TransStart Top Green qPCR SuperMix (TransGen Biotech, Beijing) was used for real-time PCR, and data collection was performed on a Bio-Rad Biosystems 7500 instrument using SYBR Green (Bio-Rad, Hercules, CA). The sequences of the forward and reverse primers are as follows:

β-actin -F: 5’-AGCGAGCATCCCCCAAAGTT-3,
β-actin-R: 5’-GGGCACGAAGGCTCATCATT-3;
TIPE-F: 5’-TTCAGGCCTCCCTCTT-TAACAATC-3,
TIPE-R: 5’-CGTTCGTGGCAGGGGTTATT-3;
DCR3-F: 5’-GCCGCTACTGCAACGTCCTC-3,
DCR3-R: 5’-GGCTGGCACTGCGTGTTCTG-3.

Relative gene expression levels were normalized to β-actin as a control.

### Flow Cytometry

The apoptosis of HCT116 cells in different treatment groups was examined by flow cytometry. Cells were seeded into six-well plates at a density of 1×10^5^ per well and incubated for 24 h. After transfection for 36 h, sFasL (100 ng/ml) was added to some groups for 24 h. The cells were then harvested, washed twice with fluorescence-activated cell sorting (FACS) wash buffer (PBS containing 1% FBS), and centrifuged at 1,000 g for 5 min. To assess cell cycle progression, 1 ml PBS was added to fully resuspend single cells, and 3 ml prechilled absolute ethanol was slowly added while gently swirling. After reaching a final concentration of 75%, cells were fixed at 4°C for 4 h. Then, the cells were washed twice with FACS wash buffer, and 500 μl propidium iodide (PI) working solution (Meilunbio, Dalian, China) was added to each cell sample; the samples were gently mixed to completely resuspend the cell pellet and then incubated at 37°C for 30 min in the dark. After staining, a CytoFlex S flow cytometer (Beckman Coulter, CA, USA) was used to analyze cell fluorescence, and FlowJo software (Stanford University, USA) was used for analysis. The cell cycle distribution is shown as the percentage of cells in G0/G1, S, G2, and M phase based on PI staining. The percentage of apoptotic cells with sub-G1 (<G1) DNA content was determined. These experiments were performed with at least three biological replicates.

### Tissue Microarray and Immunohistochemistry

Normal tissue and CRC tissue microarrays (CRC-1402) were obtained from Wuhan Servicebio Biotechnology Company (Wuhan, China). The paraffin-embedded microarray was dewaxed in an oven at 65°C overnight, dehydrated and rehydrated with xylene and a series of concentration gradients of ethanol, and subjected to heat-induced antigen recovery in a pressure cooker with a sodium citrate antigen repair solution (Maixin, Fuzhou, China) for 15 min. The samples were then blocked at room temperature with endogenous peroxidase blockers for 15 min, washed with PBST three times and incubated overnight at 4°C with anti-TIPE antibodies (1:50, Abcam, Suite Cambridge, USA) and DCR3 antibodies (1:50, BioLegend, London, United Kingdom). On the next day, the samples were washed with PBST three times, and a biotin-labeled secondary antibody was added and incubated for 30 min at room temperature. The sections were visualized with the diaminobenzidine method and then counterstained with hematoxylin. Finally, Image-Pro Plus 6.0 (Media Cybernetics, Inc., MD, USA) was used to analyze the immunohistochemical staining density and average optical (AO) density. For the negative control of the staining process, the primary antibody was omitted, while all other experimental conditions remained the same.

### Statistical Methods

Statistical Package for the Social Sciences 21.0 (SPSS 21.0), GraphPad Prism 6 and Excel were used to analyze all the results. A t-test was used for comparisons between two groups, one-way ANOVA was used for comparisons among more than two groups, and the Bonferroni method was used for comparisons between groups and within groups. Differences with *p*<0.05 were considered statistically significant (* *p*<0.05; ** *p*<0.01; *** *p*<0.001; and **** *p*<0.0001).

## Results

### Tumor Necrosis Factor-Induced Protein-8 and Decoy Receptor 3 Are Highly Expressed and Positively Correlated in Patients with Colorectal Cancer

**Figure 1:**
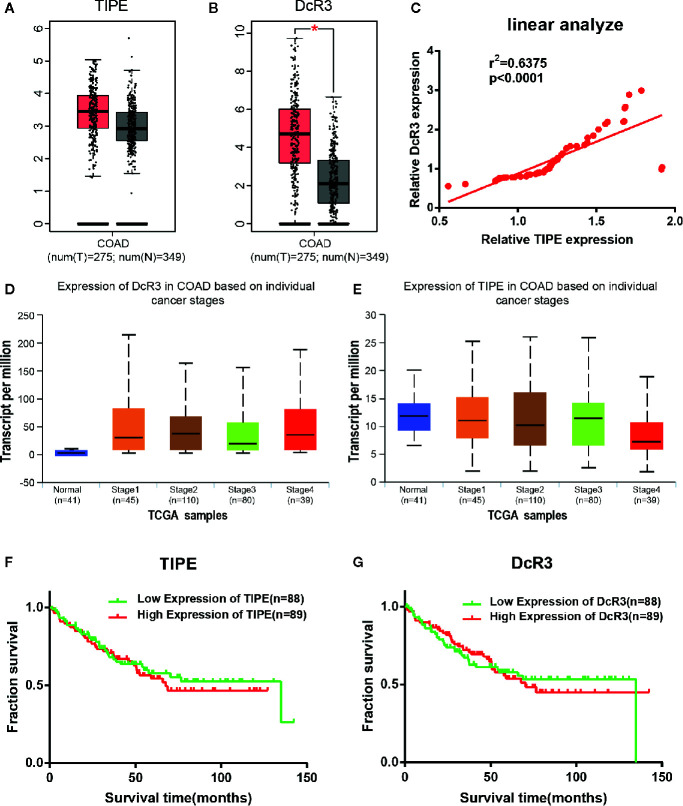
Tumor necrosis factor-induced protein-8 (TIPE) and DcR3 are highly expressed and positively correlated in colorectal cancer (CRC) patients. Box plots showing **(A)** TIPE and **(B)** DcR3 mRNA upregulation in CRC samples relative to normal samples (data downloaded from GEPIA). **(C)** The linear analysis relationship between TIPE and DcR3 showed that the proteins were positively correlated. **(D)** Relative DcR3 mRNA expression in UALCAN datasets, which included 41 normal samples and 274 samples with different stages of colorectal cancer. **(E)** Detection of relative TIPE mRNA expression in the same database. Kaplan-Meier curves for the overall survival of 177 CRC patients stratified by **(F)** TIPE and **(G)** DcR3 expression. *p < 0.05.

To investigate the role of TIPE and DcR3 in human CRC, we first analyzed the available datasets of CRC patients in the Cancer Genome Atlas (TCGA) database, and the differential expression of TIPE and DcR3 in CRC tissues and adjacent tissues was randomly validated with Gene Expression Profiling Interactive Analysis (GEPIA; http://gepia.cancer-pku.cn/index.html). The bioinformatics results showed that TIPE and DcR3 expression was significantly increased in CRC tissues compared to adjacent tissues at the mRNA level (the red area represents the tumor), and the difference in DcR3 expression was significant (**Figures 1A, B**). In addition, to build on our previous literature review, we further searched the Oncomine dataset (Gaspar) to evaluate the relationship between TIPE and DcR3, and linear correlation analysis results confirmed that the expression of TIPE and DcR3 was positively correlated (**Figure 1C**). These results suggested that TIPE and DcR3 are highly expressed in CRC and may play a vital role in the tumorigenesis and development of CRC.

To further investigate the function of TIPE and DcR3 in CRC, we continued to examine the association between their expression and clinical factors in the UALCAN (http://ualcan.path.uab.edu/index.html) database. The results confirmed that as the tumor stage gradually increased, the mRNA expression level of DcR3 increased compared with that in normal tissues (**Figure 1D**). Conversely, the expression level of TIPE decreased (**Figure 1E**) as the tumor stage increased, which may be related to the difference in tumor subtypes. In addition, we used the same dataset (GSE17536) from the Gene Expression Omnibus (GEO) database to analyze the relationship between TIPE and DcR3 and the survival of CRC patients. As shown in **Figures 1F, G**, higher expression levels of TIPE and DcR3 were associated with worse survival rates in patients, indicating that high expression levels of TIPE and DcR3 are associated with poor prognosis. This further supported that TIPE and DcR3 play a vital role in the tumorigenesis and development of CRC.

To determine whether the increase in TIPE and DcR3 expression was associated with clinical features, we collected GSE17536 data and analyzed correlations between TIPE and DcR3 expression and the sex, age, tumor grade, stage, total survival and disease-free survival of CRC patients. No significant correlations were observed in these analyses (*p*> 0.05) (**Supplementary Tables 1**, **2**).

### The Expression of Tumor Necrosis Factor-Induced Protein-8 and Decoy Receptor 3 Is Increased in Colorectal Cancer

**Figure 2:**
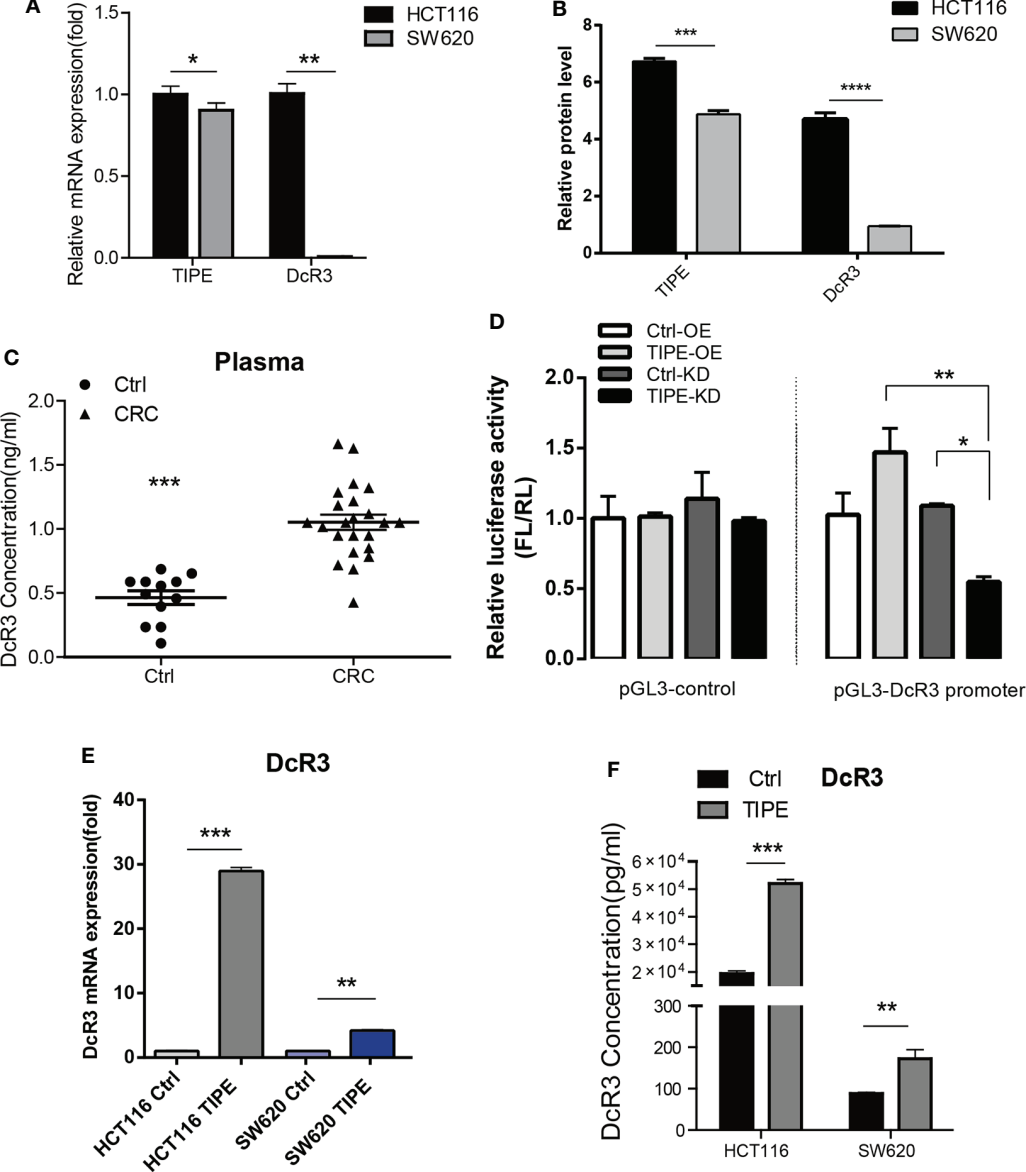
Tumor necrosis factor-induced protein-8 (TIPE) can upregulate DcR3 expression and positively regulate DcR3 transcription. **(A)** Comparison of TIPE and DcR3 mRNA expression in HCT116, and SW620 cells. TIPE and DcR3 mRNA expression was quantified by qRT-PCR and normalized in HCT116 cells. **(B)** Expression of TIPE and DcR3 in HCT116, and SW620 cells based on Western blot assays. **(C)** ELISA analysis and statistical analysis of DcR3 in a group of patients with CRC (n = 8) and healthy individuals (n = 4). **(D)** The relative luciferase activity of the DcR3 promoter was detected after TIPE overexpression and TIPE knockdown. **(E)** qRT-PCR assay of relative mRNA expression levels of DcR3 in two colon cancer cells based in TIPE overexpression or control cells. **(F)** DcR3 protein expression was detected by ELISA in supernatants of HCT116, and SW620 cells. TIPE promoted DcR3 secretion compared with that in the control group. ns, not significant, *p < 0.05, **p < 0.01, ***p < 0.001.

To verify the relationship between TIPE and DcR3 in CRC, we first selected two colon cancer cell lines, HCT116, and SW620, which are commonly used in experiments, and evaluated the expression of TIPE and DcR3 by qRT-PCR and Western blot analysis. As shown in **Figures 2A, B**, all cell lines expressed TIPE. DcR3 was low expressed in SW620 cells, and the mRNA and protein levels of TIPE and DcR3 were higher in HCT116 cells. In addition, we also collected plasma samples from patients with CRC and healthy individuals (the control group) and determined the DcR3 level in the plasma with ELISA. Plasma samples from cancer patients had significantly higher levels of DcR3 than those from the control group (**Figure 2C**). These results demonstrate that TIPE and DcR3 were highly expressed in CRC and that their protein expression patterns were consistent with their mRNA expression patterns.

DcR3 is generally difficult to detect because it lacks a transmembrane sequence, and DcR3 is a soluble protein. However, our results combined with the previous linear correlation analysis results of bioinformatics data demonstrated that TIPE was highly expressed in stage III gastric cancer and positively correlated with DcR3 and ERK1/2 expression. We hypothesized that there may be a regulatory relationship between TIPE and DcR3. Through dual-luciferase reporter gene assays, we found that overexpressing TIPE enhanced the transcriptional activity of the DcR3 promoter, whereas knocking down TIPE expression reduced the transcriptional activity of DcR3 (**Figure 2D**). We then overexpressed TIPE in two cell lines and examined whether the expression of DcR3 was altered. When the expression of TIPE increased (Supplementary **Figure 1A**), the expression of DcR3 also significantly increased compared with that in the control group (**Figures 2E, F**). In summary, these results suggested that TIPE positively regulated DcR3 transcription and that TIPE overexpression upregulated DcR3 expression.

### Tumor Necrosis Factor-Induced Protein-8 Affects Proliferation and Apoptosis by Regulating Decoy Receptor 3 Expression

**Figure 3:**
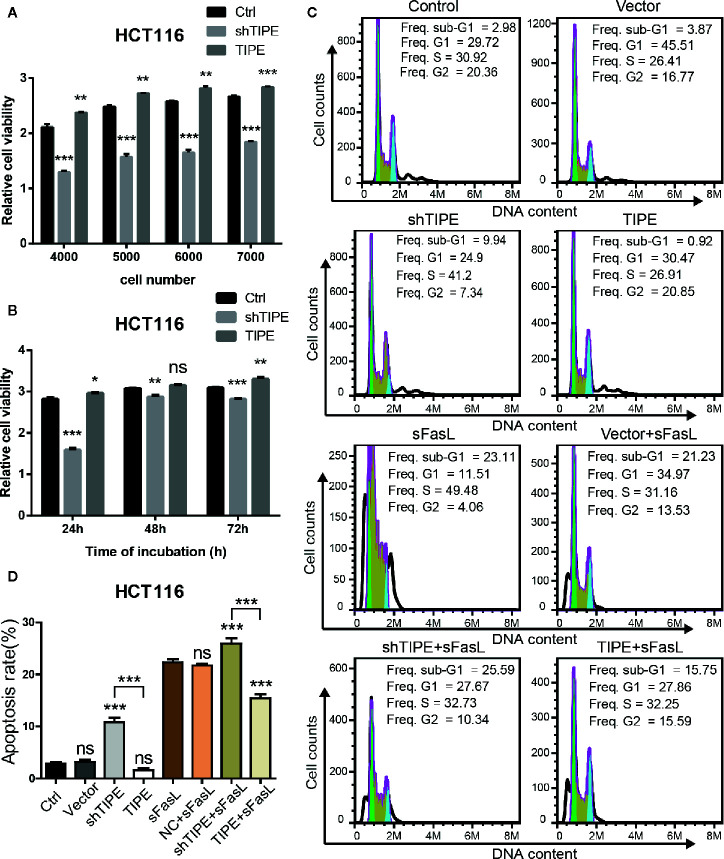
Alteration in tumor necrosis factor-induced protein-8 (TIPE) expression regulates cell proliferation and apoptosis in HCT116 cells. **(A)** TIPE was overexpressed or knocked down in HCT116 cells, and cell proliferation was measured by CCK-8 assays. **(B)** Cell proliferation assays of HCT116 cells transfected with TIPE and shTIPE after 24, 48, and 72 h of incubation. **(C)** HCT116 cells were transfected with TIPE or shTIPE for 24 (h) sFasL (100 ng/ml) was added for another 24 h, and the cells were fixed and stained with PI to analyze the DNA content with a CytoFlex S flow cytometer. The cell cycle phase (sub-G1, G1, S, and G2) is indicated. The sub-G1 phase is indicative of apoptosis. The experiment was performed three times with similar results. **(D)** Statistical analysis was used to assess the apoptosis rate of HCT116 cells after changes in TIPE,expression and sFasL treatment. ns, not significant, *p< 0.05, **p< 0.01, ***p< 0.001.

Based on the above findings, we next explored whether TIPE can affect the function of DcR3. Due to the low expression level of DcR3 and detection difficulty, we selected HCT116, which had higher expression level of DcR3 after the overexpression of TIPE, as the target cell line for our subsequent experiments. CCK-8 assays showed that cell growth declined after knocking down TIPE, while overexpressing TIPE reversed this phenomenon and promoted the proliferation of HCT116 cells. With the increase in cell number and culture time, the differences became more apparent, and they were significant compared with those in the control group (*p*<0.001) (**Figures 3A, B**). These results suggest that TIPE overexpression promoted the proliferation of colon cancer cells.

Resistance to apoptosis is considered a reason for cancer treatment failure. We first used a PI staining solution to detect apoptotic cells under normal conditions, and there was no difference in the number of sub-G1 cells between the normal group (control group) and the TIPE control group (vector group) in HCT116 cells. Subsequently, we found that the sub-G1 population was larger in the TIPE knockdown group, whereas the sub-G1 population was smaller after the overexpression of TIPE in HCT116 cells (**Figure 3C**). Statistical analysis revealed significant differences among the shTIPE group, TIPE group and control group (**Figure 3D**). Numerous studies have shown that the DcR3 protein acts as a decoy receptor, neutralizing FasL-mediated apoptosis signals. Therefore, we further exposed the cells in the above groups to sFasL (100 ng/ml) for 24 h and then detected cell apoptosis. We discovered that the sub-G1 population decreased in the TIPE-overexpressing group, while the sub-G1 and apoptotic cell populations increased in the TIPE knockout group (**Figure 3C**). Further statistical analysis confirmed that the TIPE overexpression and knockdown groups were significantly different from the control group in terms of apoptosis rates (*p*<0.001), and the TIPE overexpression group and the TIPE knockout group were also significantly different (*p*<0.001) (**Figure 3D**). Meanwhile, we also detected the secretion of DcR3 in different groups with ELISA, and the results showed that as TIPE expression increased, DcR3 also increased, and vice versa (**Supplementary Figure 1B**). These results further confirmed that the overexpression of TIPE upregulated DcR3 secretion and blocked the cytotoxic effect of FasL in HCT116 cells.

### Tumor Necrosis Factor-Induced Protein-8 Overexpression Enhances the Migration of Colon Cancer Cells

**Figure 4:**
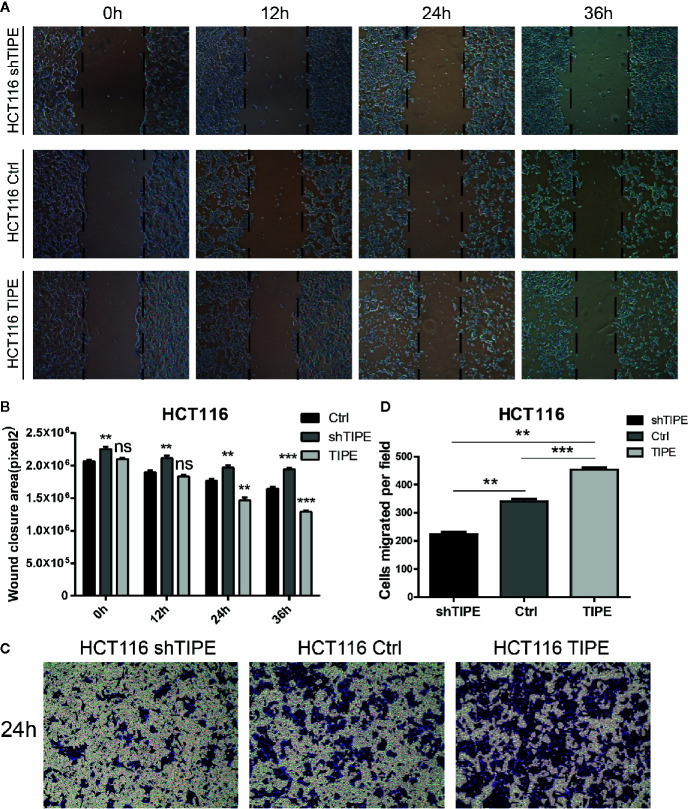
Changes in the expression of tumor necrosis factor-induced protein-8 (TIPE) affect cell migration. **(A)** After 12, 24, and 36 h incubation, in vitro wound healing assays with TIPE or shTIPE transfected HCT116 cells were performed to evaluate cell migration. **(B)** Histograms indicated the size of the wound healing area, and representative statistical results are shown. **(C)** Based on a Transwell assay, cell migration was significantly enhanced after transfection with TIPE in HCT116 cells but decreased after transfection with shTIPE. Quantitative analysis of three independent experiments is shown in **(D)**; ns, not significant, **p< 0.01, ***p< 0.001.

Since both proliferation and migration are important in tumor growth and metastasis, we further verified the effect of TIPE on the migration ability of HCT116 cells. Through a wound healing assay, we found that compared with the control group, the TIPE overexpression group showed stronger wound healing ability, especially after 24 h, while TIPE knockdown had the opposite effect (**Figure 4A**). We also performed statistical analysis of the scratch area. As shown in **Figure 4B**, the area of the TIPE knockdown group was significantly smaller than that of the control group, while the healing rate of the TIPE overexpression group significantly increased at 24 h compared with that of the control group (*p*<0.01). All these results indicated that TIPE enhanced the migration ability of colon cancer cells.

We used a Transwell assay to further evaluate the effect of TIPE on the motility of HCT116 cells. After 24 h of culture, we found that the number of adherent cells on the lower side of the Transwell chamber significantly increased in the TIPE overexpression group compared with that in the control group, while TIPE knockdown had the opposite effect (**Figure 4C**). These results again illustrate that TIPE promotes the migration of the colon cancer cell line HCT116. Statistical analysis further confirmed that there were significant differences among the three groups in terms of cell migration (**Figure 4D**). We also extracted proteins from three groups of cells and verified the effect of TIPE overexpression with Western blot analysis (**Supplementary Figure 1C**). The cell supernatant was collected, and the secretion of DcR3 was further detected with ELISA. The expression of DcR3 was increased in the TIPE overexpression group but decreased in the TIPE knockdown group (**Supplementary Figure 1D**). These results all demonstrated that TIPE overexpression promotes DcR3 secretion and cell migration.

### Tumor Necrosis Factor-Induced Protein-8 Regulates Decoy Receptor 3 Expression by Activating the Phosphatidylinositol 3-Kinase/AKT Signaling Pathway in Colorectal Cancer

**Figure 5:**
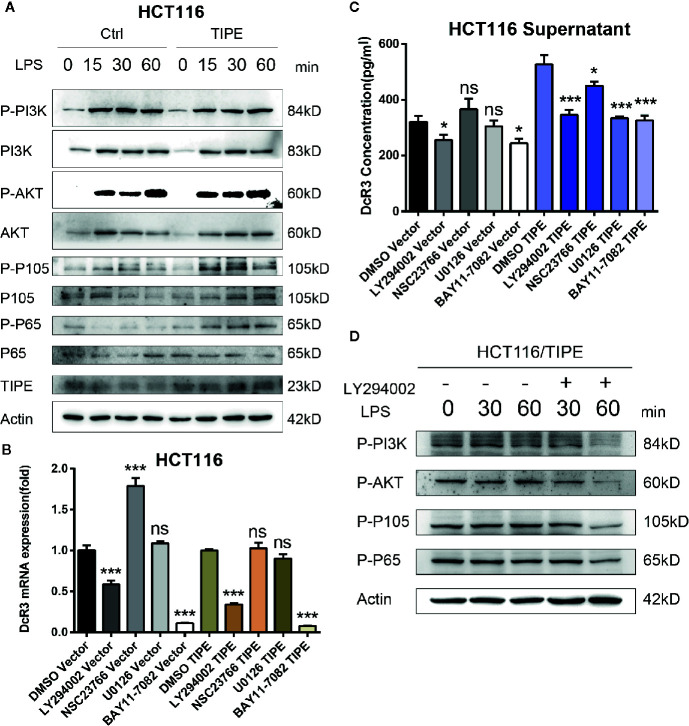
Tumor necrosis factor-induced protein-8 (TIPE)-mediated activation of the PI3K/Akt pathway is involved in the modulation of DcR3 levels in HCT116 cells. **(A)** HCT116 cells were transfected with TIPE or an empty vector (control) for 24 h with lipopolysaccharide (LPS) stimulation for the indicated time (0, 15, 30, and 60 min). Cells were harvested, and whole-cell extracts were prepared for Western blot analysis of the indicated proteins. The blots shown are representative of those obtained in three separate experiments. **(B)** HCT116 cells were cultured in 6-well plates and transfected with TIPE or an empty vector (control) for 24 h, then qRT-PCR assays were performed to measure the relative DcR3 mRNA expression of HCT116 cells after treatment with LY294002 (50 μM), NSC23766, U0126, and BAY 11-7082. Expression was normalized to that in the control cells. **(C)** The culture medium was collected, and DcR3 protein levels were measured by ELISA. **(D)** HCT116 cells were transfected with tumor necrosis factor-induced protein-8 (TIPE) after treatment with lipopolysaccharide (LPS) for the indicated time (0, 30, and 60 min), and Western blot analysis of whole-cell lysates was performed to examine the indicated proteins in HCT116 cells after treatment with or without LY294002 (50 μM). ns, not significant, *p < 0.05, ***p < 0.001.

To confirm the importance of the PI3K signaling pathway in regulating DcR3 expression in CRC after changes in TIPE expression, we stimulated the HCT116 colon cancer cell line with LPS. Through Western blot analysis, we found that in response to LPS, the expression levels of total PI3K, AKT, P105, and P65 did not change, but the phosphorylation levels of AKT, P105, and P65 increased in a time-dependent manner in response to LPS stimulation in the TIPE overexpression group compared to those in the control group (**Figure 5A**). To further examine the role of the PI3K/AKT signaling pathway in DcR3 expression, we pretreated LPS-stimulated cells with the PI3K inhibitor LY294002, Rac GTPase inhibitor NSC23766, MEK inhibitor U0126 or IκBα/NF-κB inhibitor BAY 11-7082. The results showed that compared with the DMSO group, the two groups treated with the PI3K inhibitor LY294002 or NF-κB inhibitor BAY 11-7082 had significantly decreased expression of DcR3 at the mRNA and protein levels (**Figures 5B, C**).

Then, to examine the activation of AKT and downstream NF-κB, TIPE-overexpressing HCT116 cells stimulated with LPS were further treated with the PI3K inhibitor LY294002. Although the change in phosphorylation levels after LPS stimulation was not obvious, the phosphorylation levels of PI3K, AKT, P105, and P65 in the TIPE overexpression group decreased after the PI3K inhibitor LY294002 was added and were significantly different from those in the group without inhibitor treatment (**Figure 5D**). Collectively, these results confirm that TIPE regulates DcR3 secretion by activating the PI3K/AKT signaling pathway in CRC.

### Clinical Value of Tumor Necrosis Factor-Induced Protein-8 and Decoy Receptor 3 in Colorectal Cancer

**Figure 6:**
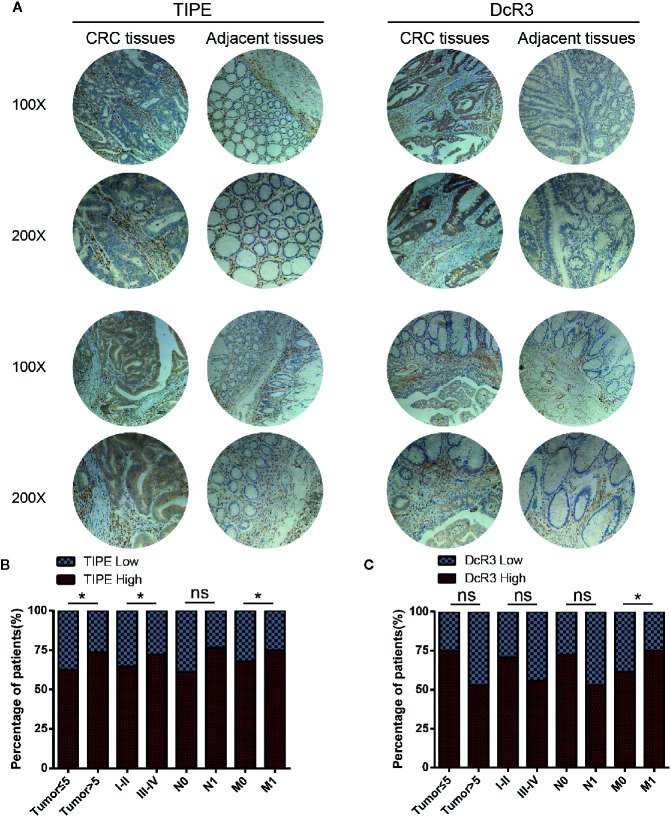
Tumor necrosis factor-induced protein-8 (TIPE) and DcR3 are upregulated in human colorectal cancer (CRC) and positively correlated with CRC metastasis. **(A)** Representative immunohistochemical detection of TIPE (left panel) or DcR3 (right panel) in tissue microarrays of CRC tissues and adjacent tissues. **(B)** Percentage of patients with high and low TIPE expression according to the following clinical parameters: tumor size (cm), tumor stage (I–II and III–IV), lymph node status (N0 or N1), and metastasis status (M0 or M1). **(C)** The percentage of patients with different expression levels of DcR3 according to different clinical parameters. ns, not significant, *p< 0.05.

To investigate the clinical value of and relationship between TIPE and DcR3 expression in CRC samples, we evaluated their expression at the protein level with immunohistochemistry. We used 35 pairs of CRC cancer tissues and corresponding adjacent tissues. Through tissue microarray analysis, we found that in the 35 pairs of samples, 24 adjacent cancer tissues were negative for TIPE expression, while the cytoplasm of tumor cells in CRC tissues showed very strong positive staining (**Figure 6A**). DcR3 staining showed that there was high expression in 22 cases (**Figure 6A**). The analysis of tumor stages showed that the expression of TIPE was increased in advanced tumors (**Figure 6B**), while the expression of DcR3 was increased in early tumors (**Figure 6C**). In addition, TIPE expression was associated with tumor size (*p*<0.05), stage of disease progression (*p*<0.05), and distant metastasis (*p*<0.05), while DcR3 expression was only associated with distant metastasis (*p*<0.05). We analyzed the survival time according to the expression of TIPE and DcR3. As the expression of TIPE and DcR3 increased, the survival time of patients decreased, but there was no significant difference (**Supplementary Figure 1E, 1F**). These results support that TIPE and DcR3 are highly expressed in CRC samples, suggesting that TIPE and DcR3 are elevated and act as oncogenes in CRC and that TIPE expression is positively correlated with CRC metastasis and poor prognosis in patients.

## Discussion

CRC is the fourth most common malignant tumor worldwide, and it has become a global threat to human life (1). Although extensive studies have been performed that focus on CRC, the detailed molecular events that occur during disease development are still unclear. More research is needed to elucidate CRC pathogenesis.

Inflammation is very closely related to the occurrence of tumors, and many factors involved in inflammation and the tumor microenvironment interact with each other to promote the occurrence and development of tumors. TIPE was the first member of the TNFAIP8 family to be identified (4). The TNFα (21) and NF-κB (22) pathways can induce the expression of TIPE, and TIPE plays an important role in inflammation and tumors (23). Numerous studies have revealed that TIPE is overexpressed in various tumors and that its overexpression is associated with clinical parameters and metastasis. In non-small-cell lung cancer (NSCLC), the expression of TIPE is downregulated *via* a decrease in EGFR levels and an increase in SNX (a key regulator of EGFR transporters) levels, which further inhibits ECL- and IGF-1-stimulated NSCLC cell migration (5). In gastric cancer patients, increased TIPE expression in tumor tissue is related to lymph node metastasis, tumor-node-metastasis (TNM) stage, and poor prognosis (24). In addition, the TIPE genotype was found to be linked to hematological malignancies, including diffuse large B-cell lymphoma (25), acute myelogenous leukemia (26), and multiple myeloma (27). TIPE is overexpressed in colon cancer and regulates cell proliferation (10). Recent studies have shown that TIPE promotes the growth and metastasis of MDA-MB-435 breast cancer cells by enhancing the expression of VEGFR2, MMP1 and MMP9 (8). Yang et al. confirmed that TIPE can promote tumor proliferation and invasion by activating Wnt signaling and inhibiting Hippo signaling in CRC (11). Our previous studies showed that TIPE was highly expressed in stage III gastric cancer and was positively correlated with DcR3 and ERK1/2 (20). Our study also found that TIPE is highly expressed in CRC, confirming that TIPE promotes angiogenesis in CRC by regulating the expression of VEGFR2. Our results revealed that TIPE regulates DcR3 expression, inhibits apoptosis, and promotes cell proliferation and metastasis.

DcR3, a member of the TNFRSF, can block apoptosis mediated by FasL, LIGHT and TL1A (28, 29). Studies have found that DcR3 is expressed at low levels in some normal tissues and in serum but overexpressed in many malignant tumors (16, 30). The serum DcR3 level in ovarian cancer is associated with tumor invasion and the number of tumor blood vessels, and the serum DcR3 level of patients is significantly decreased after tumor resection (31). Previous studies found that DcR3 mRNA levels are high in colon cancer tumors and the CRC cell lines SW480 and SW1116 (13). Our previous results showed that DcR3 is highly expressed in gastric cancer cell lines and surgically resected gastric cancer tissues. BGC823 cells were used to establish a tumor model in nude mice, and the expression of DcR3 at the inoculation site was increased during tumor development (32). Studies have confirmed that DcR3 is a predictor of 5-FU-based adjuvant chemotherapy responses in CRC (17). Yu et al. discovered that DcR3 has the potential to regulate the growth and metastasis of SW480 colon cancer cells (18). Zong et al. found that the overexpression of DcR3 in CRC can increase the risk of malignancy (19). These results suggest that DcR3 plays an antiapoptotic and proliferative role in intestinal inflammation and CRC.

In this study, we investigated the expression of TIPE and DcR3 in CRC and analyzed their correlation and relationship with CRC prognosis. We also explored the possible mechanisms by which TIPE regulates DcR3 expression. First, by searching the databases, we found that TIPE and DcR3 were highly expressed and positively correlated in CRC patients. We also collected plasma samples from CRC patients diagnosed by the Department of Gastrointestinal Surgery, Zhongshan Hospital of Xiamen University and detected significantly elevated DcR3 expression in these samples compared to control samples. Through *in vitro* cell experiments, we confirmed that TIPE and DcR3 were highly expressed in two colon cancer cell lines and that the expression levels were higher in the HCT116 cell line. Then, we overexpressed TIPE in all colon cancer cell lines and found that increased TIPE expression significantly upregulated the mRNA and protein levels of DcR3. Finally, a dual-luciferase reporter gene assay showed that the overexpression of TIPE enhanced the transcriptional activity of the DcR3 promoter.

**Figure 7:**
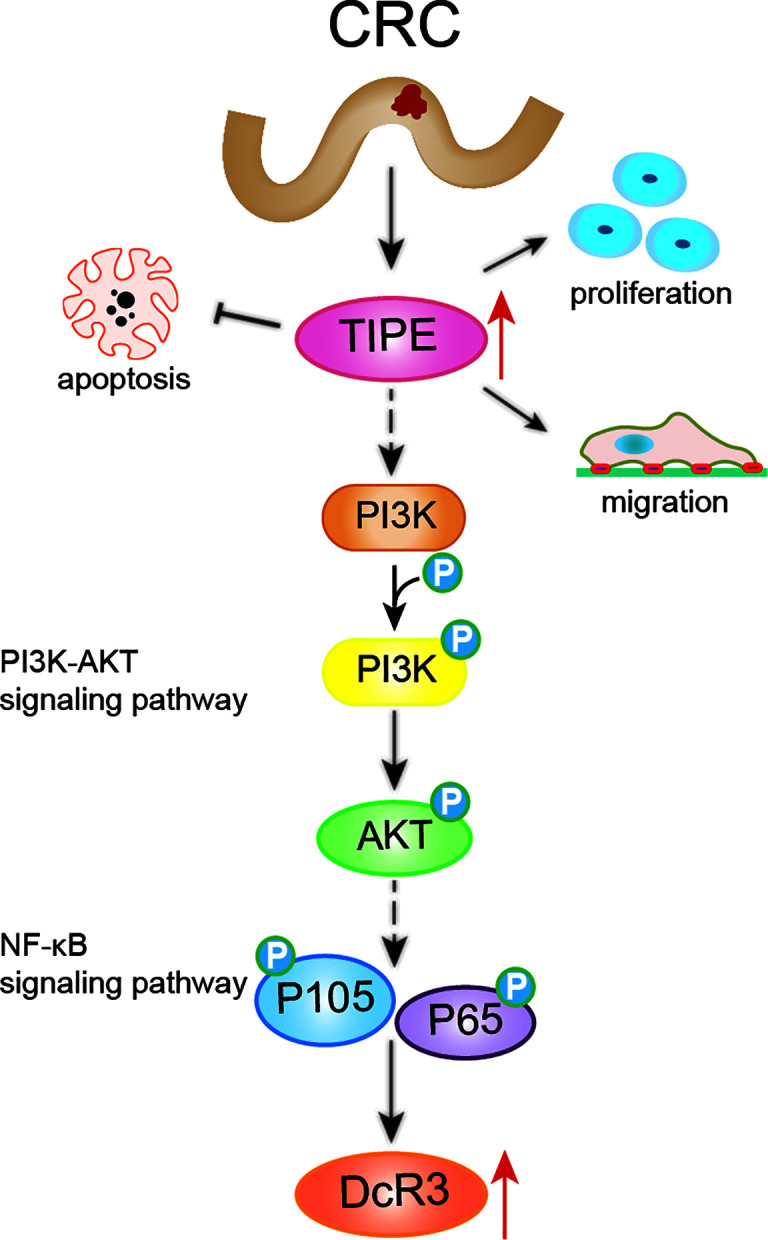
Schematic diagram representing that TIPE regulates DcR3 expression and function by activating the PI3K/AKT signaling pathway in CRC. The solid line indicates direct regulation, the dashed line indicates indirect influence.

Studies have shown that TIPE family members are the only known transfer proteins for the lipid second messengers phosphatidylinositol 4,5-diphosphate (PIP2) and phosphatidylinositol 3,4,5-triphosphate (PIP3) (33). These proteins act by regulating phosphatidylinositol 3-kinase (PI3K) and downstream mediators, such as AKT, Rac1, GSK3, ERK1/2, NF-κB, to trigger inflammation and tumorigenesis. It is widely accepted that the PI3K/AKT signaling pathway plays a crucial role in many human cancers (34). To confirm the importance of the PI3K/AKT signaling pathway in regulating DcR3 expression in CRC after changes in TIPE, we stimulated TIPE-overexpressing HCT116 colon cancer cells with LPS and found increased phosphorylation levels of AKT, P105, and p65 and NF-κB activation. Treatment with the PI3K inhibitor LY294002 significantly inhibited the phosphorylation of PI3K, AKT, P105, and P65 and decreased the expression of DcR3. These results provided preliminarily confirmation that TIPE regulates the expression of DcR3 through the PI3K/AKT signaling pathway (**Figure 7**). Functional experiments showed that the increased expression of TIPE not only upregulated the expression of DCR3 but also significantly enhanced the proliferation and migration ability of HCT116 cells and reduced FasL-induced apoptosis. Our results demonstrate that in the occurrence and development of CRC, TIPE can regulate the secretion of DcR3 and play a synergistic role in promoting tumor growth and migration. DcR3 is a soluble protein that is easy to detect, and it is associated with the occurrence, development and prognosis of tumors, so it can be used as a biomarker during tumor diagnosis and treatment response evaluation. Through tissue microarray analysis, we observed that TIPE and DcR3 act as oncogenes and have increased expression in CRC compared to normal controls. In addition, the expression of TIPE is positively correlated with CRC metastasis and poor prognosis in patients.

## Conclusions

In summary, this study revealed that TIPE and DcR3 are highly expressed in CRC and are associated with poor prognosis in patients. Furthermore, TIPE may regulate the expression of DcR3 by activating the PI3K/AKT signaling pathway in CRC, thereby promoting cell proliferation and migration and inhibiting apoptosis (**Figure 7**). Therefore, it is of great clinical significance to elucidate the pathogenesis and prognosis of CRC.

## Data Availability Statement

The datasets presented in this study can be found in online repositories. The names of the repository/repositories and accession number(s) can be found in the article/**Supplementary Material**.

## Ethics Statement

The studies involving human participants were reviewed and approved by the medical ethics committee of Zhongshan Hospital, Xiamen University. The patients/participants provided their written informed consent to participate in this study.

## Author Contributions

MZ, XQ, and GZ designed the study. MZ and YL collated the data and designed and developed the database. MZ, XQ, YY, LG, and CW conducted the experiments. MZ, YY, and HC analyzed the results. JM, ZL, and GZ provided the critical reagents. MZ and HC wrote the paper. JM, ZL, and GZ edited the manuscript and provided critical comments. All authors contributed to the article and approved the submitted version.

## Funding

This study was supported by the National Natural Science Foundation (No. 81272720 and No. 81400984), the Natural Science Foundation of Fujian Province (No. 2018J01138 and No. 2014D009), the National Health and Family Planning Commission Scientific Research Foundation-Health, the Education Cooperation Foundation (WKJ2016-2-17), and the Fujian Province Health project of Innovative Medical Talents Training and Medical Innovation Project (2017-CXB-22).

## Conflict of Interest

The authors declare that the research was conducted in the absence of any commercial or financial relationships that could be construed as a potential conflict of interest.

## Supplementary Material

Supplementary Figure 1
**(A)** qRT-PCR assaying relative mRNA expression levels of TIPE in two colon cancer cells (TIPE-overexpressing or control cells). **(B)** HCT116 cells were transfected with TIPE or shTIPE for 24 h. sFasL (100 ng/ml) was added for another 24 h, the culture medium was collected, and DcR3 levels were measured with ELISA. **(C)** Expression of TIPE in HCT116 cells transfected with TIPE, shTIPE or a control as measured by Western blot assays. **(D)** The culture medium of HCT116 cells transfected with TIPE, shTIPE or a control were collected, and DcR3 levels were measured by ELISA. **(E)** Different expressions of TIPE affected overall survival. **(F)** Compared with patients with low DcR3 levels, patients with high DcR3 levels had a higher probability of recurrence and worse overall survival. ns, not significant, **p* < 0.05, ***p* < 0.01, ****p* < 0.001.
