# Supplementary figures and images for: TIPE Regulates DcR3 Expression and Function by Activating the PI3K/AKT Signaling Pathway in CRC
Source: Front Oncol. 2021 Feb 24;10:623048. doi: 10.3389/fonc.2020.623048 (PMC7943851; doi:10.3389/fonc.2020.623048)

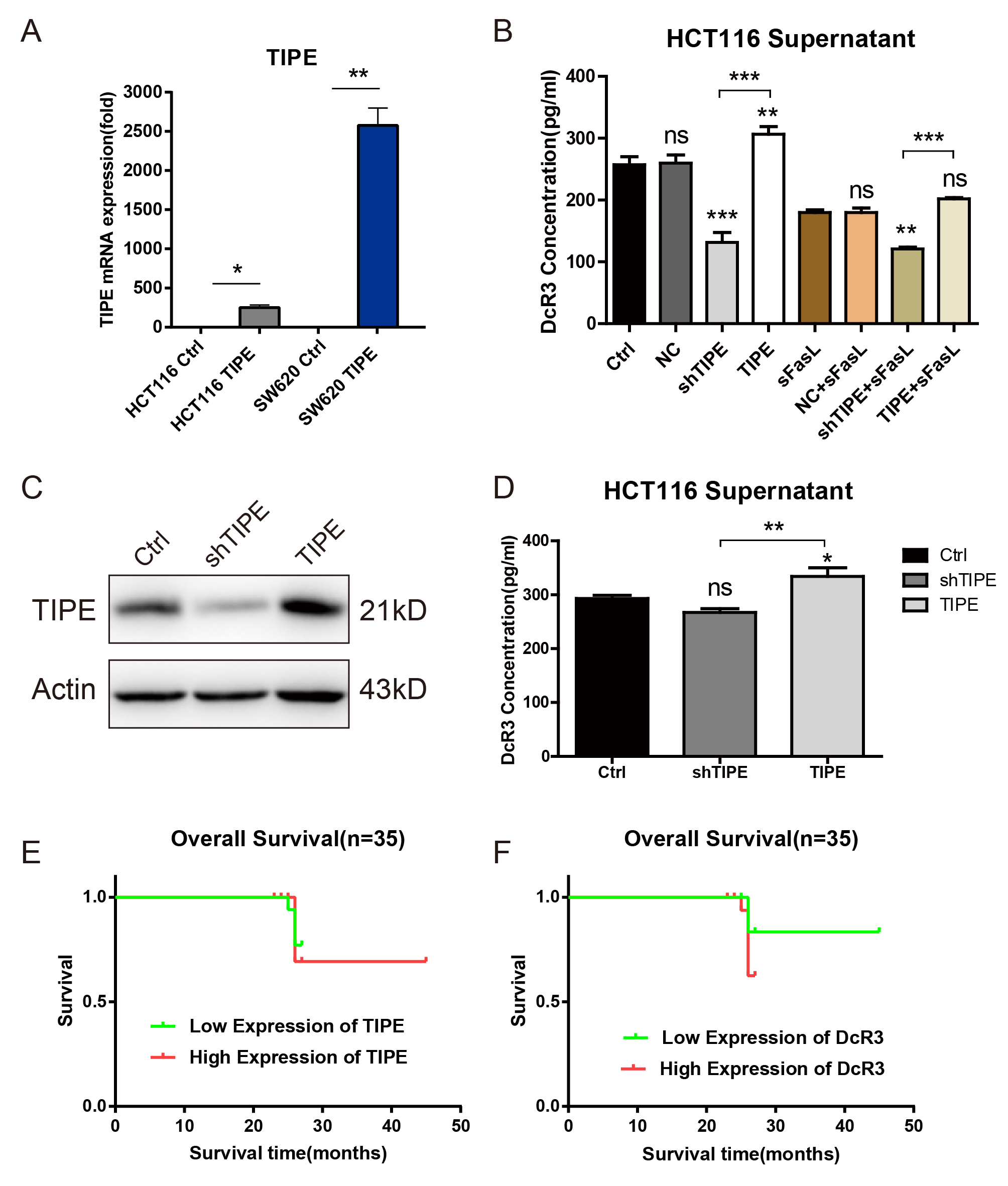

Supplement: Supplementary Figure 1 — (A) qRT-PCR assaying relative mRNA expression levels of TIPE in two colon cancer cells (TIPE-overexpressing or control cells). (B) HCT116 cells were transfected with TIPE or shTIPE for 24 h. sFasL (100 ng/ml) was added for another 24 h, the culture medium was collected, and DcR3 levels were measured with ELISA. (C) Expression of TIPE in HCT116 cells transfected with TIPE, shTIPE or a control as measured by Western blot assays. (D) The culture medium of HCT116 cells transfected with TIPE, shTIPE or a control were collected, and DcR3 levels were measured by ELISA. (E) Different expressions of TIPE affected overall survival. (F) Compared with patients with low DcR3 levels, patients with high DcR3 levels had a higher probability of recurrence and worse overall survival. ns, not significant, *p < 0.05, **p < 0.01, ***p < 0.001. [file Image_1.tif]
