# Supplementary material for: TIPE Regulates DcR3 Expression and Function by Activating the PI3K/AKT Signaling Pathway in CRC
Source: Front Oncol. 2021 Feb 24;10:623048. doi: 10.3389/fonc.2020.623048 (PMC7943851; doi:10.3389/fonc.2020.623048)
Supplement: Supplementary file 2 [file Table_1.docx]

**Supplementary Table 1. Distribution of TIPE expression in CRC patients according to clinicopathological characteristics.**

| **Characteristics** | **TIPE** | | **χ^2^** | **P value** |
| --- | --- | --- | --- | --- |
|  | Positive | Negative |  |  |
| **Gender** |  |  |  |  |
| Male | 44 | 52 | 1.2661 | 0.2605 |
| Female | 44 | 37 |  |  |
| **Age** |  |  |  |  |
| ≥66Y | 46 | 48 | 0.048951 | 0.8249 |
| <66Y | 42 | 41 |  |  |
| **Ajcc stage** |  |  |  |  |
| 1 | 14 | 10 | 3.2163 | 0.3595 |
| 2 | 26 | 31 |  |  |
| 3 | 32 | 25 |  |  |
| 4 | 16 | 23 |  |  |
| **Grade** |  |  |  |  |
| 1 | 11 | 5 | 5.522, 2 | 0.0632 |
| 2 | 60 | 74 |  |  |
| 3 | 17 | 10 |  |  |
| **Overall event** |  |  |  |  |
| Death | 38 | 35 | 0.2715, 1 | 0.6023 |
| No death | 50 | 54 |  |  |
| **Dfs event** |  |  |  |  |
| NA | 13 | 19 | 2.129, 2 | 0.3450 |
| Recurrence | 21 | 15 |  |  |
| No recurrence | 54 | 55 |  |  |
