# Supplementary material for: TIPE Regulates DcR3 Expression and Function by Activating the PI3K/AKT Signaling Pathway in CRC
Source: Front Oncol. 2021 Feb 24;10:623048. doi: 10.3389/fonc.2020.623048 (PMC7943851; doi:10.3389/fonc.2020.623048)
Supplement: Supplementary file 3 [file Table_2.docx]

**Supplementary Table 2. Distribution of DcR3 expression in CRC patients according to clinicopathological characteristics.**

| **Characteristics** | **DcR3** | | **χ^2^** | **P value** |
| --- | --- | --- | --- | --- |
|  | Positive | Negative |  |  |
| **Gender** |  |  |  |  |
| Male | 43 | 53 | 2.0361 | 0.1536 |
| Female | 45 | 36 |  |  |
| **Age** |  |  |  |  |
| ≥66Y | 49 | 45 | 0.46581 | 0.4949 |
| <66Y | 39 | 44 |  |  |
| **Ajcc stage** |  |  |  |  |
| 1 | 11 | 13 | 3.2563 | 0.3539 |
| 2 | 32 | 25 |  |  |
| 3 | 30 | 27 |  |  |
| 4 | 15 | 24 |  |  |
| **Grade** |  |  |  |  |
| 1 | 8 | 8 | 2.2872 | 0.3187 |
| 2 | 63 | 71 |  |  |
| 3 | 17 | 10 |  |  |
| **Overall event** |  |  |  |  |
| Death | 36 | 37 | 0.0080491 | 0.9285 |
| No death | 52 | 52 |  |  |
| **Dfs event** |  |  |  |  |
| NA | 13 | 19 | 1.6802 | 0.4317 |
| Recurrence | 17 | 19 |  |  |
| No recurrence | 58 | 51 |  |  |
